# Do Omega-3 Fatty Acids Have a Role in Prevention of Cardiovascular Disease?

**DOI:** 10.3389/fphys.2012.00395

**Published:** 2012-10-05

**Authors:** Thomas A. Barringer

**Affiliations:** ^1^Heart and Wellness ClinicCharlotte, NC, USA; ^2^University of North Carolina School of MedicineChapel Hill, NC, USA

As often the case within scientific research, the answer is not as straightforward as the question. In contradistinction to earlier data, recently published studies have been negative, and thus raised the question of whether supplementation with omega-3 fatty acids for prevention of cardiovascular disease is now passé. Some background knowledge is necessary to appreciate this perplexing and controversial topic.

Observational studies performed within the general population over the past 40 years have almost uniformly noted an inverse relationship between fatty fish or *n*-3 fatty acid consumption and morbidity or mortality from coronary heart disease (CHD; Kromhout et al., 1985, 2010, 2011; Dolecek, 1992; Rodriguez et al., 1996; Daviglus et al., 1997; Albert et al., 1998, 2002; Oomen et al., 2000; Iso et al., 2001; Yuan et al., 2001; Hu et al., 2002, 2003; Lemaitre et al., 2003; Mozaffarian et al., 2003; He et al., 2004; Panagiotakos et al., 2005; Mozaffarian and Rimm, 2006; Bjerregaard et al., 2010). Likewise, among studies which measured blood or tissue levels of *n*-3 fatty acids the majority have shown the same inverse correlation with cardiovascular disease events (Siscovick et al., 1995; Albert et al., 1998, 2002; Harris et al., 2007; Block et al., 2008; Park et al., 2009; Pottala et al., 2010). However, observational data can never prove cause-and-effect. Randomized clinical trials (RCT) have been designed specifically to provide a more controlled evaluation of the effects of omega-3 fatty acids treatment on adverse cardiovascular events. However, RCTs have their own limitations, especially when the interventional agent being tested is one that is available in food and consumed in varying amounts by the population participating in the trial. Among the vitamin supplement trials, for example, results derived from the whole study population have sometimes been opposite from the results from the “deficient” sub-population (Morris and Tangney, 2011; Rimm and Stampfer, 2011).

There have been about 20 published trials in which patients were randomized to a daily dose of *n*-3 fatty acid vs. placebo and who were then followed for varying intervals in order to assess some type of cardiovascular disease outcome. Most of these were performed in patients with a history of CHD.

Two recently published meta-analyses reached divergent conclusions. Kwak et al. (2012) performed a meta-analysis of 14 trials, and concluded that there was insufficient evidence of a preventive effect of omega-3 fatty acid supplements against overall cardiovascular events among patients with a history of cardiovascular disease. The authors note that there was a small reduction in cardiovascular death (RR 0.91; 95% CI: 0.84–0.99), which disappeared when one study with major methodological problems was excluded. Quality of a meta-analysis in predicated upon the quality of the individual studies and on the amount of heterogeneity among the studies. In this case, all but four of these trials had less than 600 participants, half lasted no more than a year, several were designed with angiographic endpoints, and three were performed in patients with implantable cardioverter-defibrillators (ICDs), using ICD discharges as the primary endpoint. General conclusions from such a meta-analysis are therefore highly speculative. The Agency for Healthcare Research and Quality also performed a systematic review with random effects meta-analysis (EPC Technical Papers Series, 2012). They included RCTs of at least 4 weeks duration, using EPA + DHA supplementation less than 6 g/day. The summary relative risks for all-cause mortality (17 trials, 51,264 patients) and cardiovascular mortality (14 trials, 48,500 patients) were 0.95 (95% CI: 0.89–1.01) and 0.89 (95% CI: 0.83–0.96), respectively. Whether you believe omega-3 fatty acid supplementation reduces cardiovascular mortality or not would seem to depend on which meta-analysis you prefer. However, since the studies included in both of these reviews are so diverse, a meta-analysis is not the most appropriate method to answer our question. A look at the differences among the large RCTs which were specifically designed to assess cardiovascular morbidity and mortality will shed more light, and at least clarify what are the unresolved issues.

Only eight RCTs have been of sufficient size (*n* = 2000–18,000) to provide adequate power for detecting statistically meaningful results (Kromhout et al., 1985, 2010, 2011; Burr et al., 1989; GISSI-Prevenzione Investigators, 1999; Yokoyama et al., 2007a,b; The GISSI-HF investigators, 2008; Galan et al., 2010; Rauch et al., 2010; The ORIGIN Trial Investigators, 2012). Trial designs, *n*-3 fatty acid doses, and study population characteristics were quite different. In summary, GISSI-Prevenzione Investigators (1999) and DART (1989) showed a large CV mortality benefit; JELIS (2007) showed a reduction in non-fatal CV events, but no effect on mortality; The GISSI-HF investigators (2008) showed a small mortality benefit in CHF patients; The ORIGIN Trial Investigators (2012), Omega (2010), Alpha-Omega (2010), and SU.FOL.OM3 (2010) showed no CV benefits (fatal or non-fatal) in their overall trial results. It should also be noted that there are a few clinical and experiment studies in which omega-3 fatty acids adversely altered cardiac rhythm, but effects of omega-3 fatty acids on rhythm disturbances is a separate, albeit related, topic which will not be addressed here.

The most popular explanation for the divergent outcomes is the following: The trials showing the largest benefit were older, performed in the pre-statin era, which was also a time of fewer therapeutic options in general for the patient presenting with a CVD event (i.e., virtually no revascularizations in the acute coronary syndrome setting, less anti-platelet therapies, etc). Patients participating in the more recent trials had the advantage of much more aggressive interventions as well as drug therapies that have been proven to reduce recurrent event rates, especially the statin class. Therefore, the mechanism(s) by which omega-3 fatty acids formerly improved CV outcomes may simply have been obviated by the newer better therapies.

However, the divergent outcomes of the omega-3 studies published to date also raise the possibility that only certain subgroups of patients derive a cardiovascular benefit from taking omega-3 fatty acids. Factors which may be critical in identifying the most responsive subgroups, include the following:

1. Cardiac function – In GISSI-P there was an inverse association between ejection fraction (EF) and prevention of sudden death. GISSI-HF (mean EF 33%) showed a 9% reduction in total mortality for HF patients (14% reduction in those who were compliant with medication). Patients with mildly diminished cardiac function (EF 30–45%), a group which were not systematically studied in the three negative post-MI trials (Omega, Alpha-Omega, and SU.FOL.OM3) and the one negative trial of dysglycemia patients (The ORIGIN Trial Investigators, 2012), might still be appropriate candidates for omega-3 therapy.
2. Baseline omega-3 intake – In contrast to the results from the ORIGIN trial (all participants had diabetes or pre-diabetes), the Alpha-Omega sub-study of its 1,014 diabetic post-MI patients showed that the EPA + DHA group, compared to the placebo group, had a hazard ratio (HR) of 0.51 for death from CHD (*p* = 0.04), and 0.51 for ventricular arrhythmia-related events (*p* = 0.09). In addition, the EPA + DHA + ALA group had a HR of 0.16 for ventricular arrhythmia-related events, and a HR of 0.28 for ventricular arrhythmia-related events + fatal MI (Kromhout et al., 1985, 2010, 2011). Observational data have shown not only that EPA + DHA intake up to 200–250 mg/day is associated with decreased cardiac, cardiovascular, or sudden cardiac death, but that no further reduction in fatal CHD occurs when EPA + DHA intakes exceed 200–250 mg/day (Mozaffarian and Rimm, 2006; EPC Technical Papers Series, 2012). Median baseline intake in ORIGIN was 210 mg/day, in Alpha-Omega it was 120–130 mg/day. A high-baseline intake of omega-3 fatty acids is the reason various authors have proposed for why there was no CV mortality benefit observed in the JELIS trial, whose Japanese participants had baseline blood levels of EPA + DHA roughly 10 times higher than the average American level (Mozaffarian, 2007; Yokoyama et al., 2007a,b). It also explains why the control group in JELIS had a cardiac death rate per 1000 person-years of 2.5, while in GISSI-Prevenzione it was 17. It’s likely that the baseline omega-3 intake (or more critically the tissue level, for which the estimated intake is a rough correlate) is another important factor in determining which patients will derive a benefit in prevention of (specifically) fatal CHD.
3. Baseline triglyceride (TG) levels – ORIGIN patients had a median TG level of 140 mg/dL. The Alpha-Omega subgroup of diabetic patients, in whom omega-3 fatty acid therapy reduced CHD death by ~50%, had a mean baseline value of 198 mg/dL. The JELIS subgroup of diabetic/pre-diabetic patients, who had a 22% reduced incidence in CAD events in the omega-3 arm, had a mean TG value of 175 mg/dL (Oikawa et al., 2009). The overall JELIS trial showed an inverse relationship between TG levels and benefit in CV event reduction, with no benefit in the subgroup with baseline TG levels <150 mg/dL and HDL-C >40 mg/dL. High TG and/or low HDL might possibly be a marker for who derives some CVD benefit from omega-3 supplementation.
4. Omega-3 dose – All of the large trials to date have used doses of 1 g or less per day, except JELIS, which although an open-label study, showed a 19% reduction in non-fatal CV events. Therefore, what is the most effective dose remains an unanswered question that will not be resolved by any of the studies currently in progress (ASCEND, Rishio e Prevenzione, and VITAL, all of which use <1 g/day).

At this time, it is not possible to definitively answer our original question. The factors enumerated above may or may not explain the discrepancies observed in the omega-3 trial outcomes, but each one formulates a reasonable hypothesis which needs to be addressed in future research. Otherwise, those who believe the latest research has now established that there is no role for omega-3 fatty acids in prevention of CVD may be throwing the proverbial baby out with the bathwater, and thereby deprive certain patients a potentially valuable therapy.
